# Neuroactive compounds induce larval settlement in the scleractinian coral *Leptastrea purpurea*

**DOI:** 10.1038/s41598-019-38794-2

**Published:** 2019-02-19

**Authors:** Mareen Moeller, Samuel Nietzer, Peter J. Schupp

**Affiliations:** Carl-von-Ossietzky Universität Oldenburg, ICBM, Environmental Biochemistry, Schleusenstrasse 1, 26382 Wilhelmshaven, Germany

## Abstract

Settlement of pelagic coral larvae is commonly induced by chemical cues that originate from biofilms and coralline algae. These natural settlement cues initiate signal pathways leading to attachment and metamorphosis of the coral larva. In order to investigate the settlement process and its natural inducers, it is necessary to gain a better understanding of these signal pathways. At present, the pathways and neurotransmitters involved in this signal transduction are still widely unknown. In this study, we exposed larvae of the brooding coral *Leptastrea purpurea* to five neuroactive compounds known to be present in cnidarians, and K^+^ Ions. All compounds were applied at different dilutions and settlement behavior of the larvae was documented over 48 h. Dopamine, glutamic acid and epinephrine significantly induced settlement in the coral larvae. The highest observed metamorphosis response was 54% in 10^−5^ M dopamine. Serotonin, L-DOPA and K^+^ ions did not have an influence on settlement behavior in our experiments. Exposing larvae to settlement-inducing neurotransmitters and thus bypassing the initial induction could be utilized in coral aquaculture. The active neurotransmitters should be used to further study the settlement process in *L. purpurea* in greater detail. Their role and relevance should also be assessed for other coral species as they may represent or reveal a universal inducer for coral settlement.

## Introduction

Coral reefs belong to the biologically most diverse ecosystems of our planet. By providing a multitude of ecosystem services, coral reefs generate significant revenue to economies of tropical coastal regions^1^. Despite their significant role for sustaining the livelihoods of several hundred million people, coral reefs are threatened in many ways and continue to decline and disappear at a historically unprecedented rate^2–4^. Rising sea surface temperatures caused by the ongoing climate change represent the most severe stressor^4^. Like many sessile marine animals, scleractinian corals disperse through a larval stage before settling on a suitable substrate and metamorphosing into a primary polyp. The planula larvae have sensory systems consisting of chemoreceptors which allow them to locate suitable settlement substrates^5^. Known settlement cues include light^6,7^, reef sound^8^, surface structure^9^, and chemical cues^10–13^. The presence of settlement-inducing cues triggers a sequence of processes leading to attachment and subsequent metamorphosis^14^. The apical organ, which is a sensory structure located at the aboral pole of the planula larva, is most likely involved in the metamorphic process^15^. Crustose coralline algae (CCA) and their associated bacteria are well-recognized substrates for settlement that are favored by many coral species^16,17^. Settlement-inducing CCA usually grow under conditions that are beneficial to scleractinians, i.e. exposure to high light intensity, adequate water quality and sufficient water movement. Many scientists have been investigating the origin of settlement cues and, while it could be demonstrated that certain bacteria are an important source of settlement-inducing compounds^11,18–20^, some settlement cues derive from CCA itself as well^10^. Some of the compounds produced by CCA or their associated biofilms induce settlement in very few species of coral, others seem to have an effect on a larger number of coral species. The compound 11-deoxyfistularin-3 is a species-specific inducer which triggers settlement in larvae of the coral *Pseudosiderastrea tayami*^21^. In contrast, tetrabromopyrrol (TBP), which is produced by certain *Pseudoalteromonas* strains, induces metamorphosis in a number of coral species of different families such as *Acropora millepora*, *Porites astreoides*, *Orbicella franksi* and *Acropora palmata*^11,13^. However, the ecological role that TBP plays is unclear because of its low natural concentration in the environment and the lack of larval attachment to the substrate in many corals^11,13^. Another naturally occurring compound with an impact on settlement behavior is Luminaolide, which enhances settlement rates in larvae of *Leptastrea purpurea* and several Acroporid species^22,23^.

Although a variety of natural settlement-inducing agents have been isolated and identified^11,21,23^, and gene expression during the settlement process has been described^24^, the signal pathways and neurotransmitters involved in the settlement process are still widely unknown. GLW-amide is a neuropeptide that is known to induce metamorphosis, often without attachment in *Acropora tenuis* and *A. palmata*^25–27^. It presumably “shortcuts” the normal developmental process and some of the behavioral and physiological changes normally associated with settlement^27,28^. These findings suggest that other neurotransmitters are also involved in the regulation of natural settlement behavior.

Deciphering which transmitters regulate the processes leading to attachment and metamorphosis would be a vital step to a more complete understanding of the scleractinian life cycle. By exposing larvae to neurotransmitters, the individual steps of signaling pathways leading to settlement and the involved transmitters could be identified. This has been conducted with a number of marine invertebrates like polychaetes^29–31^, gastropods^32–36^, bivalves^37–44^ and holothuroids^45,46^. The obtained knowledge found application in the commercial oyster culture where ammonia and the neurotransmitters L-DOPA, epinephrine, norepinephrine and yohimbine have been successfully used as settlement agents^47,48^.

A number of neurotransmitters and artificial compounds have been discovered to induce settlement in marine cnidarians. Larvae of the hydrozoan *Eudendrium racemosum* exhibited settlement behavior in response to serotonin^49^. Larvae of another hydrozoan, *Halocordyle disticha* settled when exposed to L-DOPA, dopamine and norepinephrine^50,51^. As reviewed in Kass-Simon & Pierobon^52^, both classical neurotransmitters, the fast-acting (acetylcholine^53,54^, GABA^55–59^, glutamate^55,56,60,61^, glycine^62–65^) and the slow-acting (catecholamines^66,67^ and serotonin^68–72^) ones, as well as neuropeptides^73–85^ play a role in the neurotransmission of cnidarians. Although many neurophysiologically active compounds have been tested on cnidarians and the corresponding receptors detected, there still is a large number of neurotransmitters whose effects on scleractinians and their role in settlement-related neurotransmission processes remain unknown.

A better understanding of the molecular basics of the settlement process of coral larvae is necessary to identify potential risks for that crucial live stage. Neuroactive environmental pollutants could interfere with the settlement of coral larvae like endocrine disruptors from sunscreen can induce calcification in larvae and cause bleaching in adults^86,87^. More knowledge about the molecules involved in this critical step in a coral’s life can enable researchers to investigate potentially problematic compounds. Since most corals only reproduce once a year an interference with the larval settlement could significantly reduce an entire cohort. A better understanding of the settlement process could also find application in coral aquaculture as well as reef restauration purposes: large numbers of coral larvae could be settled on a substrate suitable for subsequent installation in the reef.

In this study, we conducted a series of experiments with larvae of the hermatypic coral species *Leptastrea purpurea* by exposing them to several neurotransmitters. We differentiated between attachment of larvae, metamorphosis and full settlement (attachment + metamorphosis) since metamorphosis can occur without attachment of the larva. The aim of the experiments conducted for this study was to widen the knowledge about the effects of commonly occurring neurotransmitters on the settlement of scleractinian coral larvae.

## Materials and Methods

### Acquisition of coral larvae

Larvae of the brooding coral *Leptastrea purpurea* were used for the experiments. *L. purpurea* is a brooding faviid coral which is common in the waters of Guam. Its unique mode of reproduction allows constant access to larvae. In contrast to other brooding species, colonies of the population in Guam, USA, release larvae on a daily basis. *L. purpurea* can be found in shallow water and easily be collected, e.g. at Luminao reef (13°27′25.66′N 144°37′31.55′E). The colonies used to obtain larvae for this study were detached from their substrate using hammer and chisel during free-diving. Upon collection, colonies were transported to the University of Guam Marine Laboratory (UOGML) and placed in large flow-through tanks. Larval collection started after three days of acclimatization of the 150 collected colonies. Larval collection was conducted as described in Moeller *et al*.^88^ and Nietzer *et al*.^89^. In short: The corals were placed in 30 L plastic containers (30 colonies per container) with aeration but no flow-through overnight. The next morning, colonies were placed back into the flow-through tanks where they stayed during the day. The water from the collection containers was carefully filtered through a 30 µm mesh to reduce their content to 2–3 L. Larvae of *L. purpurea* contain a green fluorescent protein (GFP) and can be easily detected in the collected material using a blue fluorescent lamp and yellow barrier filters (BlueStar™, NIGHTSEA, Lexington, Massachusetts, USA). Larvae are 0.2–0.9 mm in length and can be collected with a plastic pipette without any optical magnification.

### Settlement assays

The following compounds were tested: serotonin hydrochloride, (−)-epinephrine, dopamine hydrochloride, 3,4-dihydroxy-L-phenylalanine (L-DOPA), l-glutamic acid, KCl. All pharmacological agents were obtained from Sigma-Aldrich Chemie GmbH (Munich, Germany).

Except for epinephrine, all compounds were dissolved at 1 M in MilliQ H_2_O. Epinephrine is poorly soluble in water and was dissolved in 0.005 M HCl. Serial dilutions were compiled in order to achieve the desired end concentrations by adding 50 µl of the respective solution to 5 ml of filter-sterilized seawater (FSW).

Assays were conducted in 12-well plates. Test solutions were dissolved in 5 ml FSW and replicated five times. FSW was used as negative control and small pieces of live CCA (*Hydrolithon reinboldii*) served as positive control. Due to the use of HCl solution for the epinephrine experiment, controls were conducted with both FSW as well as 0.005 M HCl. All chemicals were tested at 10^−3^ M, 10^−4^ M, 10^−5^ M, 10^−6^ M, 10^−7^ M. These concentrations have been used in several other studies investigating neurotransmitters in marine invertebrates^39,40,90,91^. In case the highest concentration did not lead to 100% mortality of the exposed larvae, chemicals were tested in higher concentrations (10^−2^ M, 10^−1^ M). The aim of this approach was to avoid missing potentially active concentrations.

**Figure 1:**
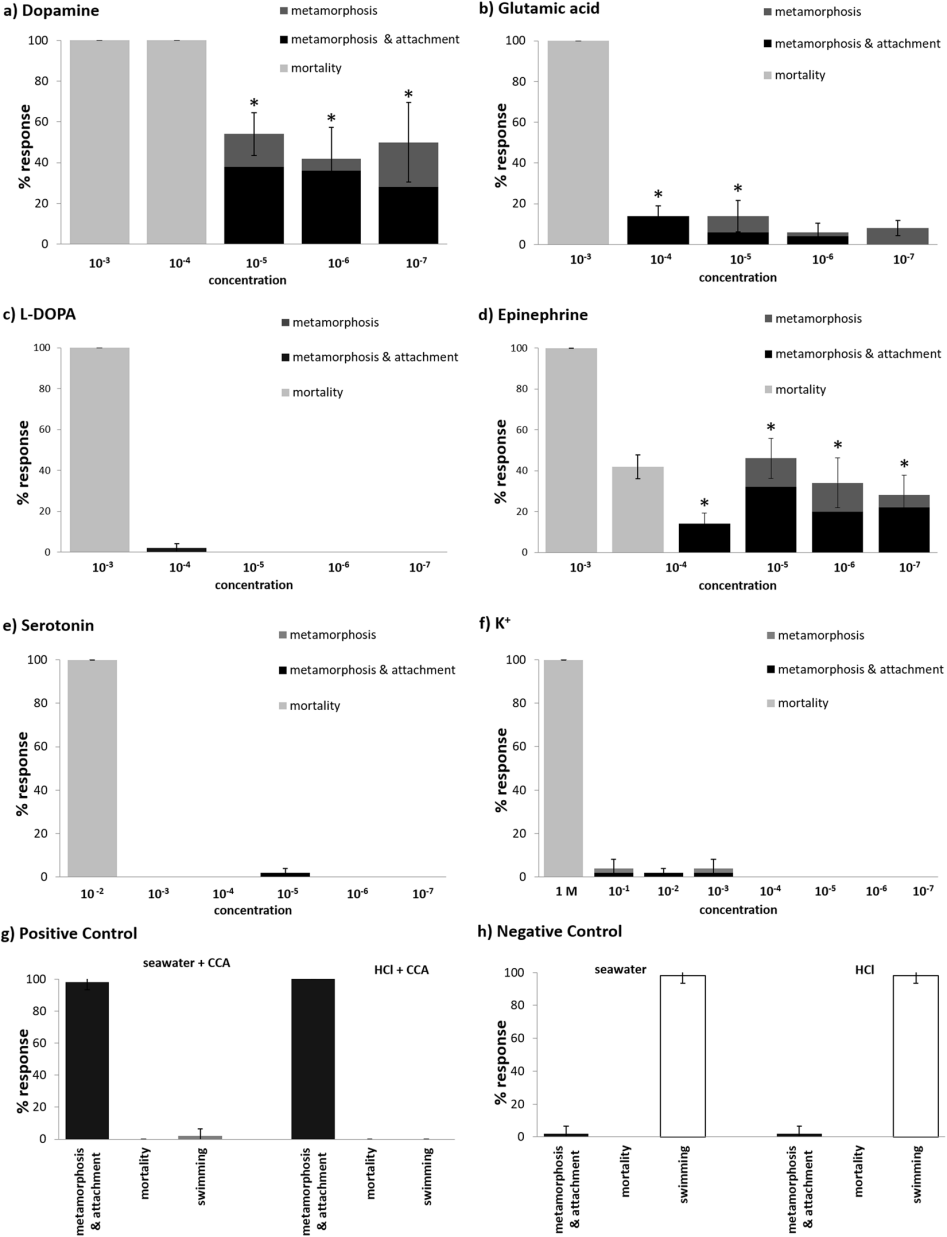
Response of *Leptastrea purpurea* larvae after 48 h to different compounds (n = 5): (**a**) dopamine (**b**) glutamic acid (**c**) phenylalanine (**d**) epinephrine (**e**) serotonin (**f**) K+ ; each tested in different molar concentrations. The percentage of metamorphosis is displayed in dark grey, metamorphosis including attachment in black, and dead larvae in light grey. Positive control (**g**) and negative control (**h**) were conducted with seawater and 0.005 M HCl. Error bars represent standard error of total metamorphosis and mortality, respectively. Asterisks represent significant metamorphosis response (p < 0.05).

Per replicate, 10 larvae were added. Larvae were examined under a dissecting microscope after 24 h and 48 h for metamorphosis, attachment and mortality. Effects are usually clearly visible after 24 h but more pronounced after 48 h: mesenteries are clearly visible in metamorphosed primary recruits whereas dead larvae start to disintegrate. The status after 48 h was used for statistical analysis and Fig. 1.

**Figure 2:**
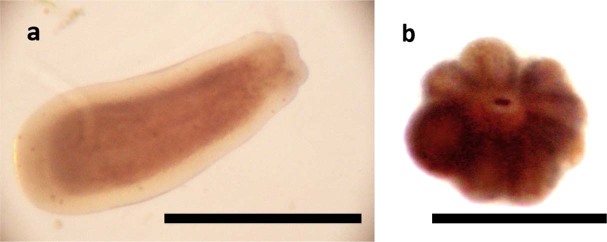
*L. purpurea* planula larva (**a**) and primary polyp (**b**). Scale bar: 0.5 mm.

We defined the documented categories as: normal swimming (no effect, Fig. 2a), metamorphosis without attachment, metamorphosis with attachment (full settlement, Fig. 2b) and mortality.

### Statistical analysis

Statistical analysis was conducted with SPSS 24 statistics.

Metamorphosis data were not normally distributed, therefore a Generalized Linear Model (GLM) with Poisson variance was applied. All data points > 0 were tested against negative control in a pairwise GLM with Wald Chi-Square Test.

## Results

Positive controls (CCA pieces) yielded 98.0 ± 4.2% full settlement (metamorphosis and attachment) (Fig. 1g). In the negative controls (FSW), 2.0 ± 4.2% full settlement was documented. Metamorphosis without attachment was not observed in the controls (Fig. 1h).

Significant effects that lead to settlement behavior (p < 0.05) were detected for dopamine, epinephrine and glutamic acid (Fig. 1a–f).

The highest metamorphosis response of 54 ± 10.4% was documented with dopamine (10^−5^ M) (p < 0.001). Stronger dilutions (10^−6^ M, 10^−7^ M) had only marginally lower effects (10^−6^ M: 42.0 ± 15.2%, p < 0.001; 10^−7^ M: 50.0 ± 19.6 p < 0.001) whereas higher concentrations (10^−3^ M, 10^−4^ M) were lethal. Glutamic acid showed low but significant metamorphosis rates at 10^−4^ M (14.0 ± 5.1%, p = 0.034) and 10^−5^ M (14.0 ± 17.3%, p = 0.034). Epinephrine had a significant effect on metamorphosis at 10^−4^ M (14.0 ± 11.4%, p = 0.034). However, the same concentration was lethal for 42.0 ± 13.0% percent of the larvae. Higher dilutions showed significant metamorphosis rates peaking at 10^−5^ M (46.0 ± 21.8%, p < 0.001) and lower rates at 10^−6^ M (34.0 ± 27.4%, p < 0.001) and 10^−6^ M (28.0 ± 22.0%, p < 0.001). Phenylalanine, serotonin and K^+^ did not have significant effects on the settlement behavior of the larvae. Lethal concentrations were 10^−3^ M for phenylalanine, 10^−3^ M for serotonin and 1 M for K^+^.

## Discussion

The conducted experiments revealed that dopamine, glutamic acid and epinephrine significantly induced settlement behavior in larvae of *Leptastrea purpurea*. All three compounds mostly induced full settlement (attachment and metamorphosis). A smaller proportion of the larvae underwent metamorphosis without attachment. As described in the introduction, GLW-amide has been documented to induce metamorphosis without attachment in larvae of *Acropora* sp., presumably by ‘shortcutting’ the natural sequences and directly inducing metamorphosis^25–28^. This suggests that GLW-amide could act as a transmitter in the metamorphic process. The results obtained in our experiments indicate that the three active compounds could also be relevant for the natural settlement process: i.e. the presence of specific settlement cues could trigger the release of these neurotransmitters and induce an upregulation of the expression of transmitter-associated genes. All tested neurotransmitters have previously been described as present in various cnidarian species (reviewed by Kass-Simon and Pierobon, 2007).

Dopamine yielded the strongest effect on settlement behavior at a concentration of 10^−5^ M: 54% of larvae underwent metamorphosis. Dopamine has been identified in both neurons and myoepithelial cells by Carlberg in *Hydra thomseni*^92^. Westfall *et al*. found dopamine and serotonin in the epidermal synapses of tentacles of the sea anemone *Exaiptasia pallida*^70^. Dopamine has also been found to play an inhibitory role in the spawning process of *Acropora* species^93,94^. Apart from the mentioned examples, dopamine has been found in many members of the cnidarian phylum as reviewed by Kass-Simon and Pierobon^52^. It is known to be a neurotransmitter in other marine invertebrates as well, and induces larval settlement in a number of invertebrates like mussels^95^, sea cucumbers^45^ and barnacles^96^. It is proposed that the modulation of motor circuits in response to environmental stimuli is one of the ancestral functions of dopamine as a signaling molecule in simple nervous systems^97^. Based on the observed effects, it seems likely that dopamine is also involved in the settlement process of coral larvae. However, given the wide range of processes that dopamine could potentially be involved in, it is possible that an exposure to high concentrations could cause off-target effects like the observed metamorphosis without attachment.

Epinephrine also induced a significant settlement response in several tested concentrations. Highest settlement response was observed at 10^−5^ M. Epinephrine was localized in the octocorallian sea pansy, *Renilla koellikeri*^98^. Norepinephrine-reactive neurons were found in the neurites, mesogloea and at the bases of its ectoderm and endoderm, and epinephrine, norepinephrine and dopamine could be identified in both neurons and myoepithelial cells^99,100^. Based on its widespread occurrence in cnidarian organisms and its strong settlement response, epinephrine may be involved in the settlement signal transduction pathway.

Dopamine and epinephrine are catecholamines that have been proposed to play an important role in the settlement process of other marine invertebrates^48,91,95,101^. Our results indicate that *L. purpurea* larval settlement is also under endogenous regulation by a catecholaminergic mechanism.

L-DOPA (3,4-dihydroxy-L-phenylalanine) had no effect on the settlement behavior of the exposed larvae. L-DOPA has been found in vesicles of ectodermal sensory cells of *Hydra* sp. and the anemone *Metridium dianthus*^92,102^. In bivalves such as oysters, epinephrine and L-DOPA are settlement inducers that find application in aquaculture^43,48^. It has been proposed that L-DOPA could be decarboxylated to dopamine and could act through dopaminergic receptors within the oyster larvae. This explains the similar effects of L-DOPA, dopamine and epinephrine in bivalve larvae^95^. Our results suggest that this mechanism does not take place in larvae of *L. purpurea*.

High concentrations of glutamate have been documented in cnidarian nematocysts^103^. In our assays, glutamic acid significantly induced settlement at 10^−4^ M. At a concentration of 10^−5^ M, glutamic acid induced full settlement but also metamorphosis without attachment. Apart from their role in the nematocysts, glutamic acids are also relevant components of immune reactions as well as feeding behavior of *Hydra* sp.^104^. Furthermore, glutamate has been shown to increase the output of both ectodermal and endodermal impulse generating systems^56^. The wide spectrum of activity in cnidarian physiology makes glutamic acids a potential signaling compound in the settlement process of coral larvae.

Despite serotonin being a known settlement inducer in different hydrozoan and scyphozoan species^49,72,105^, it did not have any significant effects on the settlement behavior of the tested anthozoan larvae in our experiment. This suggests a different signal pathway. Experiments with *Acropora millepora* exposed to CCA revealed no effect on the expression of the serotonin receptor 5htr1^28^. K^+^ ions are known to induce metamorphosis in marine invertebrates either by depolarization of cell membranes or by acting in the metamorphic signal-transduction pathway^106^. The three highest concentrations of K^+^ ions tested led to a distinct ‘flower’ shape of the larvae within the first minutes of exposure. However, larvae exposed to these concentrations were either dead or swimming normally after 24 h. The same was observed after 48 h. Although the tested concentrations yielded consistently low and insignificant settlement rates, we cannot rule out that intermediate concentrations might lead to higher rates of settlement or metamorphosis without attachment. A mere depolarization by ions might not lead to settlement in *L. purpurea*. However, other ions and concentrations should be tested to draw final conclusions.

The results obtained from these experiments revealed that three of the tested neurotransmitters could be involved in the natural settlement process. Chemical cues derived from potential settlement substrates and biofilms could induce the upregulation of transmitter expressing genes leading to attachment and metamorphosis. The influence of TBP on the regulation of GABA-expressing genes has been investigated but did not show any involvement of GABA and GABA-expressing genes in the settlement process^12^. Apart from the genes associated with the GABA system, glutamate transporter genes (vGlut) have been identified in anthozoans^107^. When more genes connected to neurophysiological agents will be located and identified in corals, a similar approach with natural settlement-inducing agents could reveal a possible involvement of other transmitters. By investigating the activity of genes coding and regulating transportation of the transmitters used in this study, speculations about their involvement in the settlement process could be verified. However, our study is limited to a single coral species and should be repeated with other species including spawning species of higher ecological relevance such as *Acropora* spp.

None of the tested compounds induced settlement as effective as the positive control which can have several reasons. It is likely that the tested concentrations were not the most effectives ones since we used a predefined dilution series. Intermediate or lower concentrations, especially in the case of dopamine, might yield settlement responses that are stronger than the documented ones. It is also possible that one single compound cannot induce settlement as well as the natural cue. Two or more compounds could have synergistic effects leading to a much stronger settlement response^21^.

A broader understanding about coral settlement could make conservation efforts more effective. A detailed knowledge about the molecules involved in the settlement process could help to identify environmental pollutants that might be interfering. For example, oxybenzone, a compound in sunscreens has recently been shown to act as an endocrine disruptor in the calcification process leading to calcified and thus unviable coral larvae^108^. Since reefs are exposed to a growing number of pollutants, knowledge about the endocrine system and signaling pathways in scleractinians is increasingly important.

Neurotransmitters could be used to induce settlement of coral larvae *ex-situ* on a large scale. If the tested compounds are also active in other scleractinian corals, mass production techniques of corals for the aquarium trade or reef restauration purposes could advance by using adequate settlement inducers such as neurotransmitters, as is common practice in the aquaculture of bivalves^47,48^. Using CCA as a settlement substrate is highly effective but comes with a variety of negative aspects, especially when applied on a large scale. Finding adequate amounts of easily accessible CCA-covered rock or dead coral branches can be challenging. If large numbers (e.g. several tens of thousands) of coral recruits are produced, several kg of CCA-covered substrate have to be removed from the environment which might impact the habitat negatively. Since natural CCA-covered substrates are usually very porous, unwanted organisms can be introduced into the rearing facilities. Aside from various algae, *Aiptasia* anemones, benthic ctenophores, ciliates as well as corallivorous snails or crabs can find their way into the tanks and reduce the survival of the coral recruits, particularly in a long-term rearing operation. Live CCA also are known competitors to juvenile corals with the ability to shed their cuticula where a coral larva or any other another epibiont has settled^109,110^. We observed this phenomenon on a regular basis. Additional to surface sloughing, an increased mucus production has been described as a protective measure from CCA against biofouling organisms^109^. These defense mechanisms of live CCA can reduce the early survival of coral recruits. Another problem could be the heterogeneity of CCA. If the corals need to settle on standardized substrates for experimental purposes, CCA chips can be a rather improper substrate. If artificial substrates such as ceramics are used for settlement, they have to be conditioned in natural seawater for several weeks in order to develop a settlement-inducing biofilm or CCA cover. In contrast, neuroactive compounds could be applied instantaneously and would likely be a cost-efficient alternative: a hypothetical application of 10^−6^ M dopamine-HCl as an inductive agent would amount to roughly $ 5 for 10.000 L of settlement medium. However, further studies should determine the most efficient concentrations and their long-term effects on settled recruits in order to assess whether neurotransmitters are a suitable way to settle large numbers of coral larvae. Although the experiments for this study were conducted with larvae obtained from 150 individual parent colonies which should eliminate phenotypic response biases, further studies with this species and a wider spectrum of compound concentrations should be conducted to corroborate the findings of this study.

The results obtained from the conducted experiments suggest that a number of neurotransmitters known to be common in cnidarians could play a role in coral larval settlement by regulating relevant biochemical signal pathways. The tested concentrations yielded a range of responses and were chosen to determine active and toxic concentrations for each of the tested neurotransmitters. Despite a moderate metamorphosis response of a maximum of 54% in dopamine, a full settlement consisting of both attachment and metamorphosis could be observed. This indicates that certain neurotransmitters could be harnessed to induce settlement of coral larvae in order to generate juvenile corals e.g. for reef restoration purposes. The identification of the settlement-inducing traits of three common neurotransmitters could be used as groundwork to further study the exact pathways involved in coral larval settlement on a molecular level. Although it is likely that the underlying pathways are of a universal nature in scleractinian corals, further research into the involved mechanisms with a range of other coral species from different families is indispensable.

## Data Availability

Data is available on figshare.com: https://figshare.com/s/f808abfdb9236c3b0d75.

## References

[1] Hughes TP (2017). Coral reefs in the Anthropocene. Nature.

[2] Burke, L., Reytar, K., Spalding, M. & Perry, A. *Reefs at risk revisite*d. *World Resouces Institute,Washington, D.C* (2011).

[3] Hughes, T. P. *et al*. Climate Change, Human Impacts, and the Resilience of Coral Reefs. **301**, 929–934 (2003). 10.1126/science.1085046 12920289

[4] Hughes TP (2017). Global warming and recurrent mass bleaching of corals. Nature.

[5] Müller WA, Leitz T (2002). Metamorphosis in the Cnidaria. Can. J. Zool..

[6] Foster T, Gilmour JP (2016). Seeing red: Coral larvae are attracted to healthy-looking reefs. Mar. Ecol. Prog. Ser..

[7] Mundy CN, Babcock RC (1998). Role of light intensity and spectral quality in coral settlement: Implications for depth-dependent settlement?. J. Exp. Mar. Bio. Ecol..

[8] Vermeij MJA, Marhaver KL, Huijbers CM, Nagelkerken I, Simpson SD (2010). Coral larvae move toward reef sounds. Plos One.

[9] Doropoulos C (2016). Characterizing the ecological trade-offs throughout the early ontogeny of coral recruitment. Ecol. Monogr..

[10] Tebben J (2015). Chemical mediation of coral larval settlement by crustose coralline algae. Sci. Rep..

[11] Tebben J (2011). Induction of larval metamorphosis of the coral Acropora millepora by tetrabromopyrrole isolated from a Pseudoalteromonas bacterium. Plos One.

[12] Siboni N (2012). Using bacterial extract along with differential gene expression in Acropora millepora larvae to decouple the processes of attachment and metamorphosis. Plos One.

[13] Sneed, J. M., Sharp, K. H., Ritchie, K. B. & Paul, V. J. The chemical cue tetrabromopyrrole from a biofilm bacterium induces settlement of multiple Caribbean corals. *Proc. R. Soc. Biol. Sci*. **281** (2014). 10.1098/rspb.2013.3086 PMC4046396 24850918

[14] Rodriguezl, S. R., Ojedal, F. P. & Inestrosa, N. C. Settlement of benthic marine invertebrates. **97**, 193–207 (1993).

[15] Tran C, Hadfield MG (2013). Localization of sensory mechanisms utilized by coral planulae to detect settlement cues. Invertebr. Biol..

[16] Johnson CR, Sutton DC (1994). Bacteria on the surface of crustose coralline algae induce metamorphosis of the crown-of-thorns starfish Acanthaster planci. Mar. Biol..

[17] Webster NS, Uthicke S, Botté ES, Flores F, Negri AP (2013). Ocean acidification reduces induction of coral settlement by crustose coralline algae. Glob. Chang. Biol..

[18] Negri AP, Webster N, Hill R, Heyward A (2001). Metamorphosis of broadcast spawning corals in response to bacteria isolated from crustose algae. Mar. Ecol. Prog. Ser..

[19] Heyward AJ, Negri AP (1999). Natural inducers for coral larval metamorphosis. Coral Reefs.

[20] Morse DE, Hooker N, Morse ANC, Jensen A (1988). Control of larval metamorphosis and recruitment in sym- patric agariciid corals. J. Exp. Mar. Bio. Ecol..

[21] Kitamura M, Koyama T, Nakano Y, Uemura D (2007). Characterization of a natural inducer of coral larval metamorphosis. J. Exp. Mar. Bio. Ecol..

[22] Kitamura, M. & Schupp, P. Luminaolide enhaces settlement in Acropors sp. *Unpubl. data* (2018).

[23] Kitamura M, Schupp PJ, Nakano Y, Uemura D (2009). Luminaolide, a novel metamorphosis-enhancing macrodiolide for scleractinian coral larvae from crustose coralline algae. Tetrahedron Lett..

[24] Strader ME, Aglyamova GV, Matz MV (2018). Molecular characterization of larval development from fertilization to metamorphosis in a reef-building coral. BMC Genomics.

[25] Erwin PM, Szmant AM (2010). Settlement induction of Acropora palmata planulae by a GLW-amide neuropeptide. Coral Reefs.

[26] Grasso LC (2011). The biology of coral metamorphosis: molecular responses of larvae to inducers of settlement and metamorphosis. Dev. Biol..

[27] Iwao, R. T. K. A cnidarian neuropeptide of the GLWamide family induces metamorphosis of reef-building corals in the genus Acropora. 127–129 (2002).

[28] Meyer E, Aglyamova GV, Matz MV (2011). Profiling gene expression responses of coral larvae (Acropora millepora) to elevated temperature and settlement inducers using a novel RNA-Seq procedure. Mol. Ecol..

[29] Pawlik J (1990). Natural and artificial induction of metamorphosis of Phragmatopoma lapidosa californica (Polychaeta: Sabellariidae), with a critical look at the effects of bioactive. Bull. Mar. Sci..

[30] Jensen R, Morse D (1988). The bioadhesive ofPhragmatopoma californica tubes: a silk-like cement containingL-DOPA. J. Comp. Physiol. B.

[31] Jensen RA, Morse DE (1990). Chemically induced metamorphosis of polychaete larvae in both the laboratory and ocean environment. J. Chem. Ecol..

[32] Morse DE, Hooker N, Jensen L, Duncan H (1979). Induction of Larval Abalone Settling and Metamorphosis By-Aminobutyric Acid and Its Congeners From Crustose Red Algae: Ii: Applications To Cultivation, Seed-Production and Bioassays; Principal Causes of Mortality and Interference. Proceedings of the World Mariculture Society.

[33] Trapido-Rosenthal H, Morse D (1985). L-α, ω-diamino acids facilitate GABA induction of larval metamorphosis in a gastropod mollusc (Haliotis rufescens). J. Comp. Physiol. B.

[34] Baloun AJ, Morse DE (1984). Ionic control of settlement and metamorphosis in larval Haliotis rufescens (Gastropoda). Biol. Bull..

[35] Wang X, Bai Y, Huang B (2010). Effects of chemical cues on larval survival, settlement and metamorphosis of abalone Haliotis asinina. Chinese J. Oceanol. Limnol..

[36] Kang KH, Kim BH, Kim JM (2004). Induction of larval settlement and metamorphosis of the abalone, Haliotis discus hannai larvae using bromomethane and potassium chloride. Aquaculture.

[37] Wassnig M, Southgate PC (2012). Effects of settlement cues on behaviour and substrate attachment of hatchery reared winged pearl oyster (Pteria penguin) larvae. Aquaculture.

[38] Nicolas L, Robert R, Chevolot L (1998). Comparative effects of inducers on metamorphosis of the Japanese oyster Crassostrea gigas and the great scallop Pecten maximus. Biofouling.

[39] Zhao B, Zhang S, Qian P-Y (2003). Larval settlement of the silver- or goldlip pearl oyster Pinctada maxima (Jameson) in response to natural biofilms and chemical cues. Aquaculture.

[40] Sánchez-Lazo C, Martínez-Pita I, Young T, Alfaro A (2012). Induction of settlement in larvae of the mussel Mytilus galloprovincialis using neuroactive compounds. Aquaculture.

[41] Yang, J., Satuito, C. G., Bao, W. & Kitamura, H. Induction of metamorphosis of pediveliger larvae of the mussel Mytilus galloprovincialis Lamarck, 1819 using neuroactive compounds, KCl, NH4Cl and organic. *Biofouling* (2008). 10.1080/08927010802340309 18701989

[42] Satuito, C., Shimizu, K. & Fusetani, N. Studies on the factors influencing larval settlement in Balanus amphitrite and Mytilus galloprovincialis. *Live Food Aquac*. 275–280 (1997).

[43] Coon SL, Bonar DB, Weiner RM (1985). Induction of settlement and metamorphosis of the pacific oyster, crassostrea gigas (Thunberg), by L-DOPA and catecholamines. J. Exp. Mar. Bio. Ecol..

[44] Coon SL, Bonar DB, Weiner RM (1986). Chemical production of cultchless oyster spat using epinephrine and norepinephrine. Aquaculture.

[45] Sun X, Li Q, Yu H, Kong L (2014). The effect of chemical cues on the settlement of sea cucumber (Apostichopus japonicus) larvae. J. Ocean Univ. China.

[46] Matsuura H, Yazaki I, Okino T (2009). Induction of larval metamorphosis in the sea cucumber Apostichopus japonicus by neurotransmitters. Fish. Sci..

[47] Helm, M. M., Bourne, N. & Lovatelli, A. Hatchery fulture of bivalves: a practical manual. *FAO Fisheries Technical Paper*. **471** (2004).

[48] Teh CP, Zulfigar Y, Tan SH (2012). Epinephrine and l-DOPA promote larval settlement and metamorphosis of the tropical oyster, Crassostrea iredalei (Faustino, 1932): An oyster hatchery perspective. Aquaculture.

[49] Zega G, Pennati R, Fanzago A, De Bernardi F (2007). Serotonin involvement in the metamorphosis of the hydroid Eudendrium racemosum. Int. J. Dev. Biol..

[50] Edwards NC, Thomas MB, Long BA, Amyotte SJ (1987). Catecholamines induce metamorphosis in the hydrozoan Halocordyle disticha but not in Hydractinia echinata. Roux’s Arch. Dev. Biol..

[51] Tran C, Hadfield MG (2012). Are G-protein-coupled receptors involved in mediating larval settlement and metamorphosis of coral planulae?. Biol. Bull..

[52] Kass-Simon G, Pierobon P (2007). Cnidarian chemical neurotransmission, an updated overview. Comp. Biochem. Physiol. A. Mol. Integr. Physiol..

[53] Kass-Simon G, Passano LM (1978). A neuropharmacological analysis of the pacemakers and conducting tissues of Hydra attenuata. J. Comp. Physiol. ϒ A.

[54] Castañeda O, Harvey AL (2009). Discovery and characterization of cnidarian peptide toxins that affect neuronal potassium ion channels. Toxicon.

[55] Delgado, L. M., Couve, E. & Schmachtenberg, O. GABA and glutamate immunoreactivity in tentacles of the sea anemone *Phymactis papillosa* (LESSON 1830). *J. Morphol*. **852**, NA–NA (2010). 10.1002/jmor.10838 20309875

[56] Kass-Simon G, Pannaccione A, Pierobon P (2003). GABA and glutamate receptors are involved in modulating pacemaker activity in hydra. Comp. Biochem. Physiol. Part A Mol. Integr. Physiol..

[57] Concas A (2016). Immunochemical Localization of GABAAReceptor Subunits in the Freshwater Polyp Hydra vulgaris (Cnidaria, Hydrozoa). Neurochem. Res..

[58] Pierobon P (1995). Biochemical and functional identification of GABA receptors in Hydra vulgaris. Life Sci..

[59] Concas a, Porcu P, Marino G, Biggio G (1998). Modulation of -Aminobutyric Acid (Gaba) Receptors and the Feeding Response By. Science (80-.)..

[60] Kass-Simon G, Scappaticci AA (2002). The behavioral and developmental physiology of nematocysts. Can. J. Zool..

[61] Scappaticci AA, Jacques R, Carroll JE, Hufnagel LA, Kass-Simon G (2004). Immunocytochemical evidence for an NMDA1 receptor subunit in dissociated cells of Hydra vulgaris. Cell Tissue Res..

[62] Pierobon P (2001). Putative glycine receptors in Hydra: A biochemical and behavioural study. Eur. J. Neurosci..

[63] Pierobon P, Tino A, Minei R, Marino G (2004). Different roles of GABA and glycine in the modulation of chemosensory responses in Hydra vulgaris (Cnidaria, Hydrozoa). Hydrobiologia.

[64] Appleton, J. R. Effects of Proline and Glycine on the Cnidocyte Discharge of Hydra magnipapillata Effects of Proline and Glycine on the Cnidocyte Discharge of Hydra (2015).

[65] Pierobon P (2012). Coordinated modulation of cellular signaling through ligand-gated ion channels in Hydra vulgaris (Cnidaria, Hydrozoa). Int. J. Dev. Biol..

[66] Kolberg KJ, Martin VJ (1988). Morphological, cytochemical and neuropharmacological evidence for the presence of catecholamines in hydrozoan planulae. Development.

[67] Elofsson R, Falck B, Lindvall O, Myhrberg H (1977). Evidence for new catecholamines or related amino acids in some invertebrate sensory neurons. Cell Tissue Res..

[68] Lentz TL, Barrnett RJ (1962). The effect of enzyme substrates and pharmacological agents on nematocyst discharge. J. Exp. Zool..

[69] Lentz TL, Barrnett RJ (1963). The role of the nervous system in regenerating hydra: The effect of neuropharmacological agents. J. Exp. Zool..

[70] Westfall JA, Elliott SR, MohanKumar PS, Carlin RW (2000). Immunocytochemical evidence for biogenic amines and immunogold labeling of serotonergic synapses in tentacles of Aiptasia pallida (Cnidaria, Anthozoa). Invertebr. Biol..

[71] Mayorova TD, Kosevich IA (2013). Serotonin-immunoreactive neural system and contractile system in the hydroid Cladonema (Cnidaria, Hydrozoa). Invertebr. Neurosci..

[72] Mayorova T, Kach J, Kosevich I (2014). Pattern of serotonin-like immunoreactive cells in scyphozoan and hydrozoan planulae and their relation to settlement. Acta Zool..

[73] Grimmelikhuijzen CJP (1985). Antisera to the sequence Arg-Phe-amide visualize neuronal centralization in hydroid polyps. Cell Tissue Res..

[74] Feld, N. & Republic, H. F. Arg-Phe-amide-like Peptides in the Primitive Nervous Systems of Coelenterates. **6**, 477–483 (1985). 10.1016/0196-9781(85)90417-6 2870476

[75] Darmer, D. *et al*. NH2 neuropeptides in Hydra magnipapillata. **412**, 403–412 (1998). 10.1042/bj3320403 PMC1219495 9601069

[76] Schmutzler C, Darmer D, Diekhoff D, Grimmelikhuijzen CJP (1992). Identification of a Novel Type of Processing Sites in the Precursor for the Sea-Anemone Neuropeptide Antho-Rfamide (Less-Than-Glu-Gly-Arg-Phe-Nh2) From Anthopleura-Elegantissima. J. Biol. Chem..

[77] Schmutzler C, Diekhoff D, Grimmelikhuijzen CJ (1994). The primary structure of the Pol-RFamide neuropeptide precursor protein from the hydromedusa Polyorchis penicillatus indicates a novel processing proteinase activity. Biochem. J..

[78] Marlow HQ, Srivastava M, Matus DQ, Rokhsar D, Martindale MQ (2009). Anatomy and development of the nervous system of Nematostella vectensis, an anthozoan cnidarian. Dev. Neurobiol..

[79] Takeda N (2018). Identification of jellyfish neuropeptides that act directly as oocyte maturation-inducing hormones. Development.

[80] Takahashi, T. & Hatta, M. The Importance of GLWamide Neuropeptides in Cnidarian Development and Physiology. **2011** (2011). 10.4061/2011/424501 PMC3268022 22312460

[81] Moosler A, Rinehart KL, Grimmelikhuijzen CJP (1996). Isolation of Four Novel Neuropeptides, the Hydra-RFamides I – IV, from Hydra magnipapillata. Biochem. Biophys. Res. Commun..

[82] Moosler A, Rinehart KL, Grimmelikhuijzen CJP (1997). Isolation of Three Novel. Neuropeptides, the Cyanea- RFamides I – III, from Scyphomedusae..

[83] Leviev, I. & Grimmelikhuijzen, C. J. P. Molecular cloning of a preprohormone from sea anemones containing. **92**, 11647–11651 (1995). 10.1073/pnas.92.25.11647 PMC40459 8524821

[84] Takahashi T (1997). Systematic isolation of peptide signal molecules regulating development in hydra: LWamide and PW families. Proc. Natl. Acad. Sci. USA.

[85] Yum S (1998). A novel neuropeptide, Hym-176, induces contraction of the ectodermal muscle in Hydra. Biochem. Biophys. Res. Commun..

[86] Downs CA (2014). Toxicological effects of the sunscreen UV filter, benzophenone-2, on planulae and *in vitro* cells of the coral, Stylophora pistillata. Ecotoxicology.

[87] Danovaro, R. *et al*. Sunscreens Cause Coral Bleaching by Promoting Viral Infections. **116**, 441–447 (2008). 10.1289/ehp.10966 PMC2291018 18414624

[88] Moeller M, Nietzer S, Schils T, Schupp PJ (2017). Low sediment loads affect survival of coral recruits: the first weeks are crucial. Coral Reefs.

[89] Nietzer S, Moeller M, Kitamura M, Schupp PJ (2018). Coral Larvae Every Day: Leptastrea purpurea, a Brooding Species That Could Accelerate Coral Research. Front. Mar. Sci..

[90] Alfaro AC, Young T, Bowden K (2014). Neurophysiological control of swimming behaviour, attachment and metamorphosis in black-footed abalone (Haliotis iris) larvae. New Zeal. J. Mar. Freshw. Res..

[91] Young T, Alfaro AC, Sánchez-Lazo C, Robertson J (2015). Putative involvement of adrenergic receptors in regulation of mussel (Perna canaliculus) larval settlement. Mar. Biol. Res..

[92] Carlberg M (1992). Localization of dopamine in the freshwater hydrozoan Hydra attenuata. Cell Tissue Res..

[93] Taira J, Higa I, Tsuchida E, Isomura N, Iguchi A (2018). Neurotransmitters in hermatypic coral, Acropora spp., and its contribution to synchronous spawning during reproductive event. Biochem. Biophys. Res. Commun..

[94] Isomura N, Yamauchi C, Takeuchi Y, Takemura A (2013). Does dopamine block the spawning of the acroporid coral Acropora tenuis?. Sci. Rep..

[95] He J (2017). Larval settlement and metamorphosis of the invasive biofouler, Mytilopsis sallei, in response to ions and neuroactive compounds. Mar. Biol. Res..

[96] Zega G (2007). Settlement of the barnacle Balanus improvisus: The roles of dopamine and serotonin. Ital. J. Zool..

[97] Barron AB, Søvik E, Cornish JL (2010). The Roles of Dopamine and Related Compounds in Reward-Seeking Behavior Across Animal Phyla. Front. Behav. Neurosci..

[98] Anctil M, Germain G, LaRivire L (1984). Catecholamines in the coelenterate Renilla koellikeri. Cell Tissue Res..

[99] Pani AK, Anctil M, Umbriaco D (1995). Neuronal Localization and Evoked Release of Norepinephrine in the Cnidarian Renilla koellikeri. J. Exp. Zool..

[100] Anctil M, Hurtubise P, Gillis MA (2002). Tyrosine hydroxylase and dopamine-β-hydroxylase immunoreactivities in the cnidarian Renilla koellikeri. Cell Tissue Res..

[101] Zega G, Pennati R, Groppelli S, Sotgia C, De Bernardi F (2005). Dopamine and serotonin modulate the onset of metamorphosis in the ascidian Phallusia mammillata. Dev. Biol..

[102] Carlberg M, Mons N, Geffard M, Nässel DR (1989). l-DOPA and fmrfamide immunoreactivity in the tentacular nerve plexus of the sea anemone Metridium senile. Comp. Biochem. Physiol. Part C, Comp..

[103] Weber JP (1990). (y-glutamic Acid)s are the Major Constituents of Nematocysts in Hydra (Hydroxoa, Cnidaria). J. Biol. Chem..

[104] Bellis SL, Grosvenor W, Kass-Simon G, Rhoads DE (1991). Chemoreception in Hydra vulgaris (attenuata): initial characterization of two distinct binding sites for l-glutamic acid. BBA - Biomembr..

[105] McCauley DW (1997). Serotonin plays an early role in the metamorphosis of the hydrozoan Phialidium gregarium. Dev. Biol..

[106] Hadfield, M., Meleshkevitch, E. & Boudko, D. The apical sensory organ of a gastropod veliger is a receptor for settlement cues. *Biol. Bull*. 67–76 (2000). 10.2307/1542804 10707814

[107] Oren M, Brikner I, Appelbaum L, Levy O (2014). Fast neurotransmission related genes are expressed in non nervous endoderm in the sea anemone Nematostella vectensis. Plos One.

[108] Downs CA (2016). Toxicopathological Effects of the Sunscreen UV Filter, Oxybenzone (Benzophenone-3), on Coral Planulae and Cultured Primary Cells and Its Environmental Contamination in Hawaii and the U.S. Virgin Islands. Arch. Environ. Contam. Toxicol..

[109] Littler MM, Littler DS (1999). Epithallus sloughing: A self-cleaning mechanism for coralline algae. Coral Reefs.

[110] McCook LJ, Jompa J, Diaz-Pulido G (2001). Competition between corals and algae on coral reefs: A review of evidence and mechanisms. Coral Reefs.

